# Analysis of coastal cod (*Gadus morhua* L.) sampled on spawning sites reveals a genetic gradient throughout Norway’s coastline

**DOI:** 10.1186/s12863-018-0625-8

**Published:** 2018-07-09

**Authors:** Geir Dahle, María Quintela, Torild Johansen, Jon-Ivar Westgaard, François Besnier, Asgeir Aglen, Knut E. Jørstad, Kevin A. Glover

**Affiliations:** 1 Institute of Marine Research (IMR), Postbox 1870, N-5817 Bergen, Norway; 2 Institute of Marine Research (IMR), Postbox 6404, N-9019 Tromsø, Norway; 3 Department of Biology, University of Bergen, Bergen, Norway

**Keywords:** Fishery, Otolith, Population, Fish, Outlier, NCC

## Abstract

**Background:**

Atlantic cod (*Gadus morhua* L.) has formed the basis of many economically significant fisheries in the North Atlantic, and is one of the best studied marine fishes, but a legacy of overexploitation has depleted populations and collapsed fisheries in several regions. Previous studies have identified considerable population genetic structure for Atlantic cod. However, within Norway, which is the country with the largest remaining catch in the Atlantic, the population genetic structure of coastal cod (NCC) along the entire coastline has not yet been investigated. We sampled > 4000 cod from 55 spawning sites. All fish were genotyped with 6 microsatellite markers and Pan I (Dataset 1). A sub-set of the samples (1295 fish from 17 locations) were also genotyped with an additional 9 microsatellites (Dataset 2). Otoliths were read in order to exclude North East Arctic Cod (NEAC) from the analyses, as and where appropriate.

**Results:**

We found no difference in genetic diversity, measured as number of alleles, allelic richness, heterozygosity nor effective population sizes, in the north-south gradient. In both data sets, weak but significant population genetic structure was revealed (Dataset 1: global F_ST_ = 0.008, *P* < 0.0001. Dataset 2: global F_ST_ = 0.004, *P <* 0.0001). While no clear genetic groups were identified, genetic differentiation increased among geographically-distinct samples. Although the locus Gmo132 was identified as a candidate for positive selection, possibly through linkage with a genomic region under selection, overall trends remained when this locus was excluded from the analyses. The most common allele in loci Gmo132 and Gmo34 showed a marked frequency change in the north-south gradient, increasing towards the frequency observed in NEAC in the north.

**Conclusion:**

We conclude that Norwegian coastal cod displays significant population genetic structure throughout its entire range, that follows a trend of isolation by distance. Furthermore, we suggest that a gradient of genetic introgression between NEAC and NCC contributes to the observed population genetic structure. The current management regime for coastal cod in Norway, dividing it into two stocks at 62°N, represents a simplification of the level of genetic connectivity among coastal cod in Norway, and needs revision.

**Electronic supplementary material:**

The online version of this article (10.1186/s12863-018-0625-8) contains supplementary material, which is available to authorized users.

## Background

The Atlantic cod (*Gadus morhua* L.) is a demersal fish found in the northern waters across the Atlantic. Due to its large size and historical high abundance, cod has formed the basis of some of the most commercially important fisheries in the Atlantic for centuries. However, a legacy of over-exploitation, potentially exacerbated by climate-driven changes in distribution, has left several cod populations depleted [1]. In turn, this has also resulted in the decline of many of the commercially significant fisheries [2, 3]. The best-known example of this is the total collapse of the northern cod fishery off Newfoundland which fell from ~ 800,000 tons around 1970 to < 1000 tons by 1992 [4].

Cod is one of the best studied marine fishes, and as for many marine species where it has been investigated, statistically significant spatial population genetic structure has been observed (reviewed by [5–7]). The first studies of population genetic structure of cod were conducted in the 1960’s using haemoglobin (e.g. [8–11]) and transferrin [12]. Shortly after, population genetic studies were performed using allozymes [13–15], mtDNA [16, 17], Pantophysin (Pan I) [18–21] and microsatellites [7, 22–28]. More recently, single nucleotide polymorphisms (SNPs) [29–34] and full-genome sequencing [35, 36] have been used to investigate the evolutionary relationships among cod populations. While not all studies on cod have revealed statistically significant population genetic structure [15, 16, 37], the majority have, and collectively, they reveal a species displaying population genetic differentiation throughout its native range.

Studies have revealed genetic differences between cod sampled on the eastern and western sides of the Atlantic [7, 30, 38], and between cod sampled in different ecosystems: in the Baltic vs. North Sea [25], North Sea vs. Norwegian coast [24], Canadian coastal vs. oceanic [38, 39], coastal and oceanic in Gulf of Maine [40], and Greenland coastal vs. offshore with link to the Icelandic offshore cod [41, 42]. Also, genetic differences have been observed between cod sampled in different areas within countries, including the North Sea [23], within UK [7], Iceland [43], North America [38, 40], and Norway (North East Arctic Cod (NEAC) vs. Norwegian Coastal Cod (NCC)) [27, 31, 32, 44]. Finally, even on small spatial scales, such as among neighbouring fjords in Norway, genetic differences have been observed [24, 26]. These genetic studies therefore demonstrate that the species consists of multiple populations displaying varying levels of connectivity.

In addition to spatial genetic structure, genetic differences have been observed among cod “ecotypes” displaying stationary and long-distance migratory behaviours. For example, large genetic differences have been reported between NCC and NEAC, which display stationary and migratory behaviours respectively [29, 31, 32]. The same has also been observed between migratory North Sea cod (NSC) and coastal cod in southern Norway [45], between migratory and stationary in Iceland [31, 46], and Canada [47]. Furthermore, the population genomic approaches [48] utilised in many of the most recent studies have also revealed large genomic inversions, notably in linkage groups 1, 2 and 7 that are assumed to be responsible for much of this strong divergence between ecotypes [29, 32]. However, genomic islands of divergence, probably also caused by genomic inversions, have also been reported between low-salinity adapted cod in the Baltic, and the North Seas [49].

Despite the considerable number of population genetic studies on cod, there are still a number of remaining questions. This is the case for Norway, which is the country with the largest remaining commercial catch of cod in the Atlantic. In Norway, coastal cod are currently divided into two management stocks, defined as NCC north of 62°, and coastal cod south of 62° (from hereon we refer to both of these components as NCC for simplicity). While the NEAC stock is currently at a high level [50, 51], NCC is depleted and a rebuilding plan has been recommended by ICES [51, 52]. Importantly, while studies of NCC sampled in neighbouring fjords have been conducted in limited geographic areas [24, 26], population genetic structure has not been investigated in detail along the entire Norwegian coastline spanning.

**Fig. 1:**
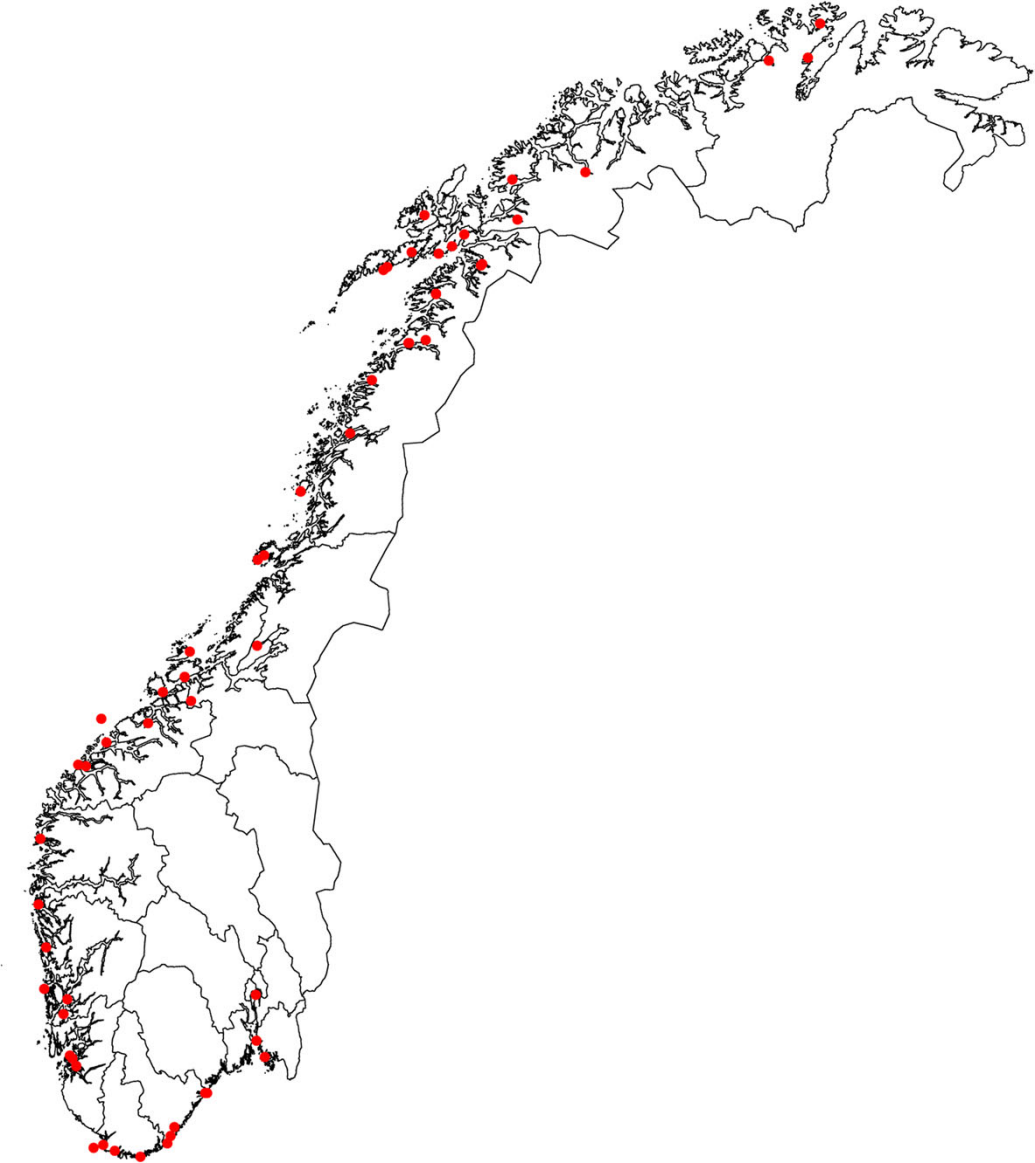
Location of the sampling sites along the Norwegian coastline (Figure produced specifically for this manuscript)

In 2002, a project was initiated to map the population genetic structure of NCC along the entire Norwegian coast. This included sampling cod from 55 spawning locations spanning the Russian to Swedish borders with Norway (Fig. 1). At the same time, biological data and brood-stock fish were sampled [53], some of which have formed the basis of common-garden experiments [54, 55]. Here, we present the results of the genetic analysis based upon data from microsatellite loci and the Pan I locus.

## Methods

### Sampling

In the period from 2002 to 2007, 4422 cod were collected from 55 locations along the coast of Norway (Fig. 1). Eleven of those sites were sampled more than once during the six-year period. Sampling was conducted during the spawning season (late winter and early spring) by taking samples from the catch of local fishermen. All of these fish were collected at known spawning sites. Most of the cod were sampled using gill nets, although for some individuals around the Lofoten islands, demersal trawls were also used. Biometry data such as length, weight and sex of all individuals were recorded, and the gonads were visually inspected to determine the stage of maturation. Fin clips from all the individuals were taken and stored in 96% ethanol prior to DNA extraction.

Although genetic analyses have also been used for identification of NEAC and NCC in Norwegian fisheries [56, 57], in routine Norwegian stock assessment north of 62°, cod are assigned to NEAC and NCC by otolith category. Otoliths from all the fish sampled in this study were analysed to determine age, age at first time of spawning and the number of spawning periods, as well as to identify individuals as NEAC or NCC. Otolith type 1 and 2 is assigned NCC whereas 4 and 5 are assigned to NEAC, as described for the first time by Rollefsen [58] and modified by Berg & Albert [59]. Assignment otolith category is in strong agreement with results from genetic markers [27, 59], and particularly Pan I. At this marker, NCC show a high frequency of the Pan I AA genotype and NEAC show a high frequency of the Pan I BB genotype [26]. In the present study, all individuals with otoliths belonging to types 4 and 5, were removed from the majority of the statistical analyses in order to exclude any NEAC from the samples, which could influence estimates of NCC population genetic structure (76 such individuals were removed in total).

### Genotyping

DNA extraction was performed using the Qiagen DNeasyH96 Blood & Tissue Kit; each of which contained two or more negative controls. All 4346 individuals were genotyped at six microsatellite loci: Gmo2, Gmo3, Gmo34, Gmo35, Gmo132 and Tch11 [60–62], together with the Pantophysin locus Pan I [18]. In addition, a subset of 1295 individuals from 17 of the sites were genotyped for an extra set of nine microsatellites (bringing the total microsatellite loci up to 15): GmoC18, GmoC20 [63]; GmoG13, GmoG18 [64]; GmoG25, GmoG40, GmoG43, GmoG45 [64], and Tch22 [62]. Thus, the present study includes two overlapping data sets: one with all 4346 individuals genotyped for six microsatellites and Pan I (55 locations, hereon referred to as Dataset 1), and the other with a sub-set of individuals 1295 individuals genotyped for 15 microsatellites and Pan I (17 locations, hereon referred to as Dataset 2). The PCR conditions are available from the authors upon request. PCR products were analysed on an ABI3130XL sequencer (Applied Biosystems), whereas Pan I was genotyped on 2.5% MetaPhore gels. Microsatellite alleles were scored using GeneMapper v4.0 (Applied Biosystems).

### Statistical analysis

Statistical analyses were conducted separately for microsatellites and Pan I. The total number of alleles, and allelic richness, were both calculated with MSA [65], whereas observed (H_O_) and unbiased expected (uHe) heterozygosity, inbreeding coefficient (F_IS_) and deviations from the expected Hardy-Weinberg distribution, were computed with GenAlEx [66]. Possible linkage (LD) between all locus pairs per population was tested using the program GENEPOP on the web [67] with significance based on the Markov chain method with 10,000 dememorizations, 20 batches and 5000 iterations per batch. Effective population size (Ne) per sample was computed using LDNE [68], implementing the threshold values of lowest allele frequency of 0.05 and 0.01. Where applicable, signification was corrected by multiple comparisons by sequential Bonferroni correction [69] implemented in the calculator developed by Justin Gaetano (2013).

To test if loci deviated from neutrality, outlier analyses were conducted with LOSITAN [70] under a stepwise model and the following settings: 1000000 simulations, 99.5% confidence interval, forced mean F_ST_, and with a 0.01 false discovery rate. Genetic differentiation among sampling sites was tested using the Analysis of Molecular Variance (AMOVA) as well by pairwise F_ST._ Both analyses were implemented in ARLEQUIN v.3.5.1.2 [71] and significance was calculated after 10,000 permutations. Likewise, hierarchical AMOVA was conducted by pooling populations according to geographic areas depicted in Table 1.

**Table 1:** Summary statistics for Dataset 1 (66 samples genotyped at six microsatellites): geographic region the sampling site belongs to, number of sample, sample name, total sampling size (i.e. NEAC and NCC), sampling size for NCC (i.e. individuals with otoliths 1 and 2); % of NEAC in the sample (i.e. individuals with otoliths 4 and 5); number of alleles; AR, allelic richness (based on a 25 diploid individuals); observed (Ho) and unbiased expected heterozygosity (He), inbreeding coefficient (F_IS_), number of deviations from Hardy-Weinberg equilibrium (HWE) and from Linkage Disequilibrium (LD) at α = 0.05

Region	No	Sample	N (total)	N (NCC)	% NEAC	N alleles	Ar	Ho	uHe	F_IS_	No dev HWE	No dev LD	Ne	
													0.05	0.01
Finnmark	1	Magerøysundet_2002	70	69	1.4	69	9	0.570	0.610	0.038	1	1	254.6	415.9
Finnmark	2	Smørfjord_2002	63	59	6.3	71	9.5	0.602	0.671	0.069	3	1	687.1	∞
Finnmark	3	Smørfjord_2003	78	74	5.1	70	9.2	0.617	0.644	0.028	2	0	∞	494.6
Finnmark	4	Repparfjord_2003	59	58	1.7	67	9.3	0.638	0.694	0.053	0	1	318.3	∞
Troms	5	Balsfjord_2002	94	94	0.0	78	9.3	0.638	0.681	0.042	2	0	1476.5	∞
Troms	6	Balsfjord_2003	81	81	0.0	73	9.1	0.656	0.662	−0.009	0	1	149.8	1080.3
Troms	7	Tranøybotn_2004	102	102	0.0	83	9.8	0.691	0.703	0.007	2	1	∞	∞
Troms	8	Tranøybotn_2005	74	74	0.0	74	9.7	0.631	0.683	0.050	3	0	∞	1037.4
Troms	9	Gratangen_2004	53	53	0.0	67	9	0.657	0.667	0.015	0	0	269.9	285.1
Troms	10	Gratangen_2005	61	61	0.0	72	9.5	0.634	0.666	0.016	2	0	∞	1203
Troms	11	Gratangen_2006	62	62	0.0	75	9.8	0.621	0.674	0.073	0	2	101.2	347.9
Lofoten	12	Eidsfjord_2003	68	68	0.0	77	10.2	0.645	0.694	0.072	2	0	∞	∞
Lofoten	13	Eidsfjord_2004	77	75	2.6	74	9.6	0.689	0.700	0.014	0	1	852.3	∞
Lofoten	14	Valberg_2006	56	43	23.2	67	9.7	0.624	0.680	0.051	1	0	∞	∞
Lofoten	15	Henningsværstraumen_2006	39	39	0.0	70	10.3	0.688	0.732	0.036	0	1	589	∞
Lofoten	16	Austnesfjord_2002	57	45	21.1	65	9.3	0.685	0.706	0.040	0	1	214.6	∞
Lofoten	17	Bresja_2006	40	40	0.0	64	9.7	0.675	0.702	0.017	0	1	260.7	4814.9
Lofoten	18	Kanstadfjord_2006	78	75	3.8	75	9.7	0.636	0.695	0.083	1	1	325.3	∞
Lofoten	19	Fiskerfjord_Tjeldsund_2006	47	47	0.0	68	9.6	0.660	0.688	0.022	1	0	126.1	131.5
Lofoten	20	Stefjord_2002	52	52	0.0	72	9.9	0.638	0.706	0.090	1	3	430.8	426.7
Lofoten	21	Tysfjord_2003	36	36	0.0	62	9.5	0.671	0.707	0.028	0	1	287.7	∞
Lofoten	22	Baldkjosen_Nordfolda_2004	57	56	1.8	73	10.1	0.708	0.712	−0.017	1	0	∞	∞
Helgeland	23	Hopen_2005	46	44	4.3	71	10.2	0.633	0.689	0.095	1	0	248.5	∞
Helgeland	24	Saltenfjord_Hopen_2004	63	63	0.0	73	9.8	0.616	0.690	0.113	1	1	∞	∞
Helgeland	25	Skjerstadfjord_Valnesfjord_2005	89	88	1.1	79	9.8	0.661	0.711	0.068	1	1	508.7	∞
Helgeland	26	Skjerstadfjord_Valnesfjord_2006	47	47	0.0	74	10.2	0.691	0.711	0.021	1	1	464.5	∞
Helgeland	27	Glomfjord_Ørnes_2004	46	46	0.0	68	9.8	0.623	0.683	0.091	2	0	125.1	154.6
Helgeland	28	LilleSjona_2004	78	78	0.0	76	9.8	0.673	0.685	0.003	1	1	∞	3959.9
Helgeland	29	LilleSjona_2005	83	83	0.0	76	9.7	0.596	0.691	0.127	2	0	∞	∞
Helgeland	30	Vega_2002	38	38	0.0	65	9.7	0.645	0.710	0.103	1	0	∞	∞
Helgeland	31	Vega_2003	83	81	2.4	75	9.6	0.652	0.684	0.053	2	1	1406.6	2103.4
Helgeland	32	Langesundet_2004	64	64	0.0	77	10.4	0.661	0.708	0.061	0	0	∞	∞
Helgeland	33	Vikna_2003	79	79	0.0	79	9.9	0.700	0.711	0.033	1	2	34,313.7	7039.7
Helgeland	34	Verrabotn_2005	72	72	0.0	75	10	0.620	0.697	0.094	1	2	1373.1	2636.2
Helgeland	35	Verrabotn_2006	60	60	0.0	69	9.3	0.639	0.695	0.071	2	3	∞	2992.9
Møre	36	Frøya_2004	63	63	0.0	76	10.3	0.720	0.736	0.027	0	0	∞	∞
Møre	37	Hitra_Laksovik_2004	85	85	0.0	83	10.3	0.694	0.724	0.032	1	0	693	∞
Møre	38	Smøla_2003	79	78	1.3	78	10.1	0.722	0.730	0.015	1	1	∞	∞
Møre	39	Vinjefjord_2004	99	99	0.0	85	10.1	0.694	0.723	0.032	0	1	727.1	4552.7
Møre	40	Batnesfjord_2004	56	56	0.0	76	10.3	0.670	0.696	0.026	1	1	∞	5604.4
Møre	41	Buagrunn_2004	48	35	27.1	61	9.4	0.700	0.718	−0.004	1	0	∞	∞
Møre	42	Midsund_2004	69	69	0.0	80	10.5	0.681	0.723	0.055	0	1	∞	548.8
Møre	43	Godøy_2004	76	65	14.5	73	9.8	0.679	0.705	0.018	3	1	∞	366.1
Møre	44	Borgunfjord_2004	132	129	2.3	85	10.2	0.703	0.735	0.047	1	1	∞	6957.6
Møre	45	Borgunfjord_2005	87	85	2.3	75	9.8	0.665	0.719	0.056	1	0	∞	∞
Vestlandet	46	NV_Bømlo_2006	57	57	0.0	70	9.7	0.722	0.736	−0.006	1	1	186.2	472.6
Vestlandet	47	Nærøysund_2007	92	92	0.0	76	9.6	0.699	0.727	0.028	1	1	∞	659
Vestlandet	48	Byrknesøy_Gulen_2007	27	27	0.0	59	9.7	0.660	0.734	0.119	3	3	45.6	56.1
Vestlandet	49	Kolltveitosen_2004	55	55	0.0	74	10.2	0.721	0.732	−0.001	1	1	∞	∞
Vestlandet	50	Halsenøy_Kloster_2006	93	93	0.0	85	10.5	0.720	0.738	0.009	1	0	378.6	∞
Vestlandet	51	Ålfjord_2004	89	89	0.0	72	9.5	0.700	0.715	0.000	1	1	232.8	258.3
Vestlandet	52	Boknafjord_v/Finnøy_2006	59	59	0.0	71	9.9	0.701	0.723	0.021	1	1	1343.1	1083.3
Vestlandet	53	Finnøy_2007	35	35	0.0	72	11.1	0.710	0.745	0.031	1	1	∞	∞
Vestlandet	54	Tau_2007	25	25	0.0	60	10	0.667	0.709	0.062	1	0	120	269
Sørlandet	55	Siragrunnen_2007	80	80	0.0	79	10.2	0.673	0.723	0.055	1	0	1368.4	∞
Sørlandet	56	Lista_nordvest_2007	36	36	0.0	66	10.1	0.731	0.758	0.053	1	0	255.5	129.5
Sørlandet	57	Farsund_2005	46	46	0.0	69	9.9	0.630	0.710	0.106	1	1	∞	∞
Sørlandet	58	Mandal_2005	67	67	0.0	73	9.8	0.657	0.724	0.061	2	4	∞	∞
Sørlandet	59	Lillesand_ytre_2005	62	62	0.0	68	9.5	0.672	0.712	0.041	0	0	∞	1507.4
Sørlandet	60	Vallesverdfjord/Lillesand_2005	45	45	0.0	70	10.1	0.670	0.727	0.049	1	2	124.1	∞
Sørlandet	61	Lillesand_2007	77	77	0.0	84	10.4	0.716	0.724	−0.005	2	0	961.6	∞
Sørlandet	62	Søndeledfjorden_Nordfjord_Risør_2005	88	88	0.0	83	10.1	0.693	0.727	0.044	2	0	∞	∞
Sørlandet	63	Risør_ytre_2005	78	78	0.0	80	9.9	0.667	0.728	0.070	2	1	∞	∞
Sørlandet	64	Oslofjord_2005	85	85	0.0	73	9.3	0.700	0.713	−0.003	0	2	626.5	3948.3
Sørlandet	65	Larkollen_2005	95	95	0.0	81	9.9	0.688	0.725	0.030	2	0	∞	∞
Sørlandet	66	Hvaler_2005	85	85	0.0	78	10.1	0.686	0.731	0.039	1	1	1229.2	∞

Effective population size (Ne) was calculated using two values of lowest allele frequency (0.05 and 0.01). Negative values of Ne have been replaced into the infinite symbol (∞). Sites have been ordered from north to south and information about the geographic region where they are placed is provided. The total number of deviations from HWE dropped from 73 to 25 after Bonferroni correction, and from 54 to 7 at LD, respectively

Several parameters such as the number of alleles, allelic richness, allele frequency, Ho, uHe and pairwise F_ST_ were tested for trends in the geographic north-south gradient using the non-parametric Kendall measure of rank correlation [72], which measures the similarity of the orderings of the data when ranked by north-south gradient or by the value of the variable tested [73], and implemented in the R Package ‘Kendall’ [74].

Population genetic structure and connectivity were investigated using two approaches. First, through BARRIER 2.2 software [75], which aims to reveal genetic barriers between populations using Monmonier’s [76] algorithm. The significance for this analyses was tested by bootstrapping 1000 matrices computed with Nei’s DA genetic distance [77]. In addition, STRUCTURE v. 2.3.4 [78] was used to identify genetic groups under a model assuming admixture and correlated allele frequencies without population information. STRUCTURE analyses were parallelised with the program ParallelStructure [79] to speed up computation time. After multiple runs of both programs, and probably due to the nature of the genetic structure observed (see results), the software failed to find clear barriers (data not presented for BARRIER). Therefore, in order to complete some of the population genetic analyses (i.e., AMOVA as described above), we subjectively chose six geographic regions for some of the hierarchical analyses, which are not advocated as Management Units.

## Results

**Fig. 2:**
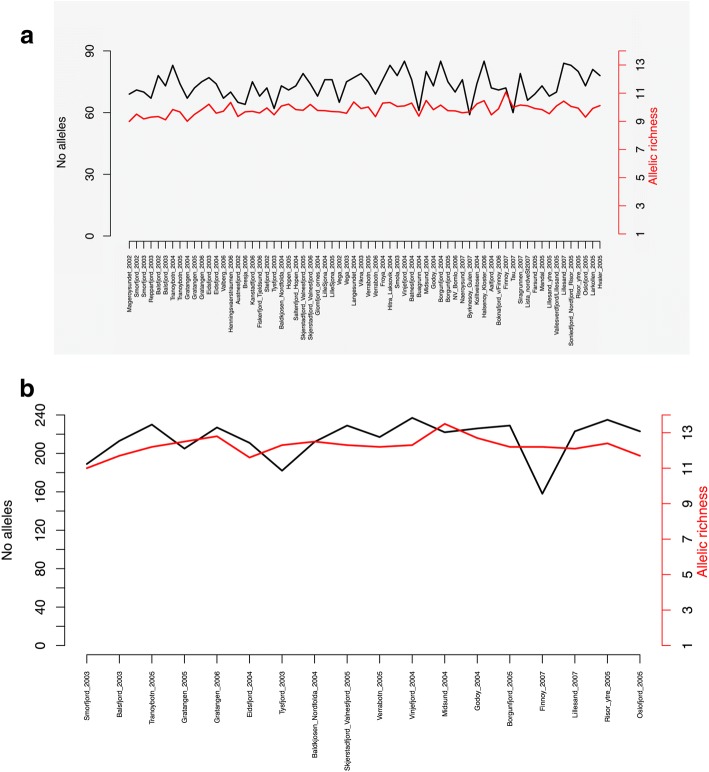
Number of alleles (black line) and allelic richness, Ar (red line) per sampling site for (**a**) Dataset 1 (6 microsatellites) and (**b**) Dataset 2 (15 microsatellites). Samples are ordered from north to south. In graph **a**), Ar experienced an increasing N-S trend (τ = 0.296, *P* = 0.00045) but not the number of alleles (τ = 0.153, *P* = 0.074); whereas in graph **b**), neither number of alleles nor Ar showed any sort of geographic trend (τ = 0.243, *P* = 0.1721; and τ = 0.0543, *P* = 0.7888, respectively)

Dataset 1 included samples from 55 locations (66 samples when temporal samples were included), made up of 25–129 individuals each, that were genotyped for six microsatellites and Pan I. Across the six microsatellites, the total number of alleles observed per sample ranged from 59 to 85, and allelic richness ranged from 9.0–11.1 (Table 1). Allelic richness displayed a trend in the north-south gradient, increasing towards the south (τ = 0.296, *P* = 0.00045) (Fig. 2a). In contrast, no trend in the number of alleles was observed on the north-south gradient (τ = 0.153, *P* = 0.074) (Fig. 2a). Observed (Ho) and unbiased heterozygosity (uHe), ranged from 0.570–0.731 and 0.610–0.758 respectively (Table 1, Additional file 1: Fig. S1a). These parameters also displayed statistically significant (albeit very weak) trends in the north-south gradient (τ = 0.363, *P* = 1.705 e-05 for Ho, and τ = 0.570, *P* < 2.22 e-16 for uHe).

Dataset 2 included a sub-set of 18 samples from 17 locations from Dataset 1, made up of 33–96 individuals each, that were genotyped with an extra suite of nine microsatellites. Across the microsatellites, a total of 158–237 alleles were observed per sample (Table 2). Neither the number of alleles (158–237), nor allelic richness (11–13.5), displayed a trend in the north-south gradient (τ = 0.243, *P* = 0.1721; and τ = 0.0543, *P* = 0.7888, respectively) (Fig. 2b). Two of the sites (Tysfjord_2003 and more importantly, Finnøy_2007) showed a lower number of recorded alleles than expected, most probably due to their low sampling sizes (*N* = 42 and *N* = 33, respectively). Neither Ho (0.632–0.734) nor uHe (0.669–0.769) showed any trend in the north-south gradient (τ = 0.281, *P* = 0.11164 and τ = 0.21, *P* = 0.23997, respectively) (Additional file 1: Figure S1b).

**Table 2:** Summary statistics for Dataset 2 (18 samples genotyped at 15 microsatellites): sample size; number of alleles; AR, allelic richness (based on 33 diploid individuals); observed (Ho) and unbiased expected heterozygosity (uHe), inbreeding coefficient (F_IS_), number of deviations from Hardy-Weinberg equilibrium (HWE) and from Linkage Disequilibrium (LD) at α = 0.05

Region	Sample	Sample size	No alleles	Ar	Ho	uHe	F_IS_	No dev. HWE	No dev. LD	Ne	
										0.05	0.01
Finnmark	Smørfjord_2003	68	189	11.0	0.650	0.728	0.095	6	4	∞	∞
Troms	Balsfjord_2003	77	213	11.7	0.703	0.737	0.029	5	3	∞	∞
Troms	Tranøybotn_2005	89	230	12.2	0.681	0.748	0.082	6	6	∞	∞
Troms	Gratangen_2005	63	205	12.5	0.669	0.696	0.023	3	1	∞	1323.2
Troms	Gratangen_2006	62	227	12.8	0.696	0.752	0.067	3	4	∞	∞
Lofoten	Eidsfjord_2004	77	211	11.6	0.691	0.753	0.072	4	8	∞	∞
Lofoten	Tysfjord_2003	42	182	12.3	0.668	0.719	0.051	3	4	∞	∞
Lofoten	Baldkjosen_Nordfolda_2004	53	212	12.5	0.734	0.763	0.029	4	2	∞	∞
Helgeland	Skjerstadfjord_Valnesfjord_2005	88	229	12.3	0.697	0.764	0.084	4	4	2338.6	3281.5
Helgeland	Verrabotn_2005	73	217	12.2	0.710	0.752	0.043	4	4	∞	∞
Møre	Vinjefjord_2004	96	237	12.3	0.711	0.768	0.066	6	4	∞	∞
Møre	Midsund_2004	67	222	13.5	0.680	0.722	0.060	4	18	451.6	377.1
Møre	Godøy_2004	64	226	12.7	0.722	0.745	0.019	4	4	∞	∞
Møre	Borgunfjord_2005	90	229	12.2	0.713	0.765	0.063	5	2	∞	5147.1
Vestlandet	Finnøy_2007	33	158	12.2	0.632	0.669	0.049	2	1	∞	∞
Sørlandet	Lillesand_2007	80	223	12.1	0.714	0.750	0.044	6	2	∞	∞
Sørlandet	Risør_ytre_2005	86	235	12.4	0.698	0.769	0.091	7	4	∞	∞
Sørlandet	Oslofjord_2005	87	223	11.7	0.705	0.744	0.043	3	4	1702.2	2914.6

Effective population size (Ne) was calculated using two values of lowest allele frequency (0.05 and 0.01). Sites have been ordered from north to south and information about the geographic region where they are placed

In the six microsatellites common to both datasets, the total number of alleles observed per locus showed extremely similar values, despite that the number of individuals genotyped displayed a 3-fold difference between the two data sets (Table 3). In five of the six microsatellite markers used in Dataset 1, global F_ST_ per locus significantly differed from zero (Table 3). In eight of the fifteen microsatellite markers used in Dataset 2, global F_ST_ per locus was significantly different from zero. The locus Gmo132 clearly displayed a much higher global F_ST_ than any of other loci in both data sets, and was reported to be under directional selection by LOSITAN (*P* = 1.0).

**Table 3:** Summary information for the markers used in this study: linkage group (LG) to which they belong to, whether the linkage group displays a known inversion, global F_ST_ (and associated *P*-value after 10,000 permutations) and number of alleles. n/a stands for “not available”

			Global F_ST_ (*P*-value)		No alleles	
Locus	LG	Inversions	Dataset 1	Dataset 2	Dataset 1	Dataset 2
Gmo2	6	No	**0.0021 (*P*** **= 0.0114)**	**0.0025 (*P*** **= 0.0324)**	23	23
Gmo3	18	No	**0.0027 (*P*** **= 0.0180)**	0.0022 (*P* = 0.1191)	13	10
Gmo34	1	Yes	**0.0047 (*P*** **< 0.0001)**	**0.0028 (*P*** **= 0.0308)**	10	10
Gmo35	20	No	0.0009 (*P* = 0.1757)	**0.0030 (*P*** **= 0.0200)**	16	16
Gmo132	7	Yes	**0.0283 (*P*** **< 0.0001)**	**0.0269 (*P*** **= 0.0000)**	52	41
Tch11	n/a	n/a	**0.0020 (*P*** **= 0.0003)**	0.0015 (*P* = 0.0788)	29	27
GmoC18	n/a	n/a		0.0002 (*P* = 0.4351)		19
GmoC20	n/a	n/a		0.0000 (*P* = 0.5175)		21
GmoG13	n/a	n/a		0.0021 (*P* = 0.1768)		18
GmoG18	n/a	n/a		*****		5
GmoG25	n/a	n/a		*****		63
GmoG40	n/a	n/a		0.0035 (*P* = 0.1111)		16
GmoG43	n/a	n/a		0.0011 (*P* = 0.1241)		30
GmoG45	n/a	n/a		**0.0056 (*P*** **= 0.0000)**		47
Tch22	n/a	n/a		*****		6
Pan I	1	Yes	**0.107 (*P*** **< 0.0001)**		2	

LG is in accordance with nomenclature from Hubert et al. [48]. Microsatellites were blasted against the gadMor2 assembly [101] by Per Erik Jorde (pers. comm)

*P*-values in boldface type were significantly different from zero

Global F_ST_ over all microsatellites was low but statistically significant in both datasets (Dataset 1, F_ST_ = 0.0075, *P* < 0.0001, and Dataset 2, F_ST_ = 0.0042, *P* < 0.0001). Global F_ST_ did not change when removing the individuals showing the genotype Pan I BB, which is the dominating genotype in NEAC and observed in very low frequency in NCC. This meant excluding 104 and 39 individuals from Datasets 1 and 2 respectively (data not shown). After excluding the microsatellite identified to be under positive selection (Gmo132), global F_ST_ decreased, but it was still statistically significant in both Dataset 1 (F_ST_ = 0.00227, *P* < 0.0001), and Dataset 2 (F_ST_ = 0.00200, *P* < 0.0001).

**Fig. 3:**
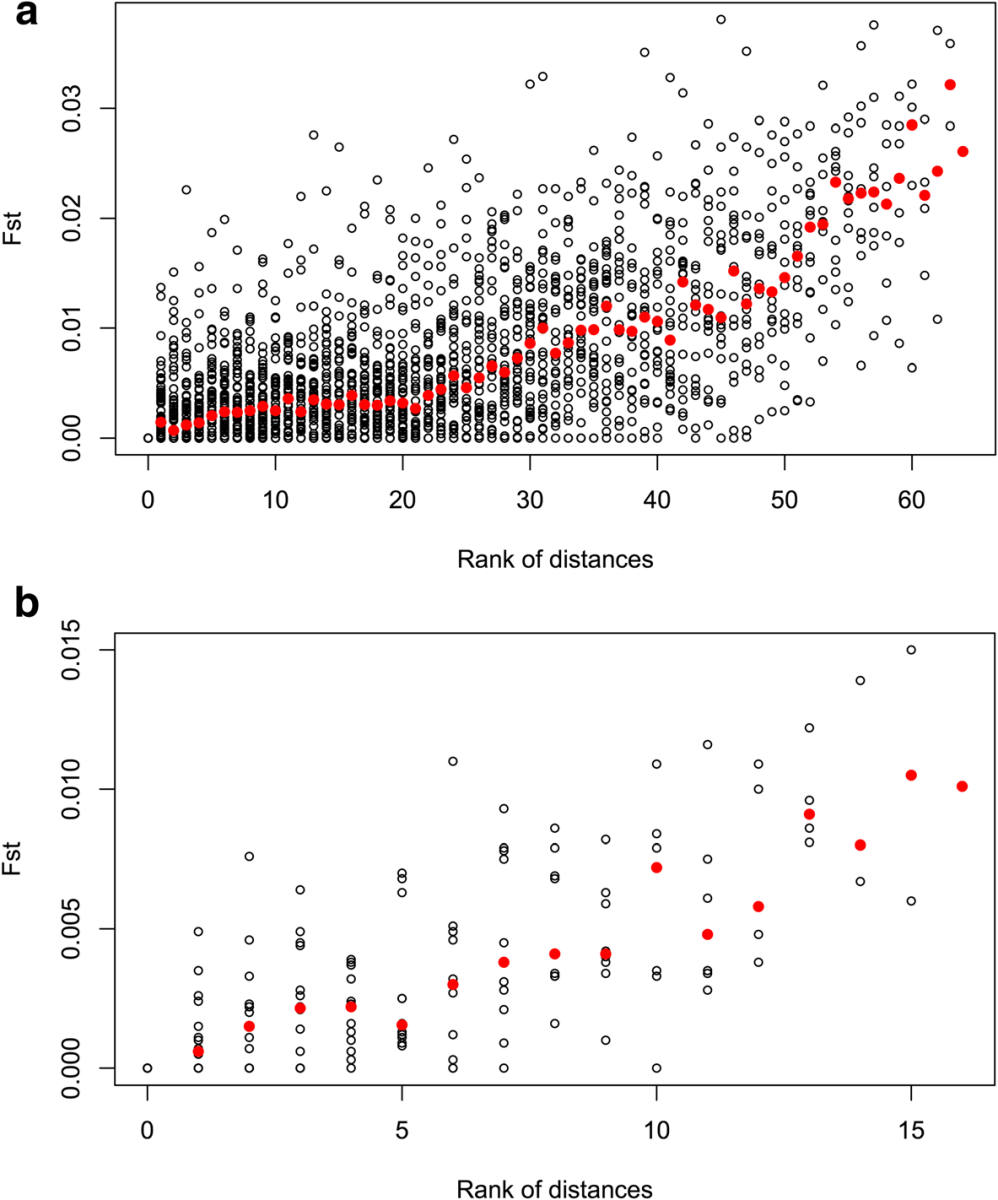
Pairwise F_ST_ for: **a**) Dataset 1 and **b**) Dataset 2. Black empty circles depict the pairwise F_ST_ for pairs of sites within the same rank of distances whereas filled red circles dots depict the median for each class. Both trends are highly significant and reveal increasing levels of differentiation correlating with the rank of distances: τ = 0.899, *P* ≤ 2.22 e-16; τ = 0.895, *P* = 1.79 e-06 and τ = 0.871, *P* < 2.22 e-16 for **a**) and **b**), respectively

The genetic matrix for Dataset 1 revealed that 2.7% of the pairwise F_ST_ comparisons within the seven geographically-determined regions were significant (which equates to 19% of the total combinations), in contrast with 50.5% of the pairwise comparisons among regions (i.e. 59% of the total, Additional file 2: Table S1). However, when using Bonferroni correction for multiple comparisons, the corrected critical value dropped to 0.00005, which involved a decrease from 2.7 to 0.1% of significant pairwise F_ST_ within regions, and from 50.5 to 20.5% among regions. Similarly, the genetic matrix for Dataset 2 revealed that 4% of the pairwise F_ST_ comparisons within the seven geographically-determined regions were significant (i.e. 24% of the total combinations), in contrast with the 55% found among regions (65.6% of the total, Table 4). The corrected critical value dropped to 0.0003 after Bonferroni correction resulting in no significant comparisons within regions, and a drop of 55 to 27% of significant pairwise F_ST_ among regions. These data suggest isolation by distance, in agreement with the fact that pairwise F_ST_ values were found to be strongly correlated with the ordering of the samples in the north-south gradient, both for Dataset 1 (τ = 0.899, *P* < 2.22 e-16) and Dataset 2 (τ = 0.895, *P* = 1.79 e-6) (Fig. 3a-b).

**Table 4:**
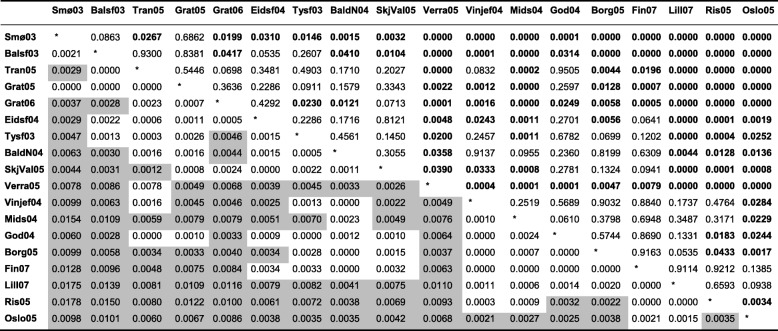
Genetic differentiation between the 18 samples in Dataset 2 (15 microsatellite markers): pairwise F_ST_ (below diagonal) and *P*-value after 10,000 permutations (above diagonal)

Likewise, site names have been shortened (see Summary table for full names). Initially, some 59% of the comparisons (90 out of 153) were significantly different from zero but, after Bonferroni correction (corrected critical value = 0.0004), the percentage reduced to 27% (41 out of 153)

F_ST_ with associated *P*-values < 0.05 have been shaded in grey to easy the reading

Hierarchical AMOVAs were conducted for datasets 1 and 2 using the aforementioned geographic approach to define regions. In both cases, the three levels of division (among regions, among populations within regions and within populations) showed significant values (Table 5). The differentiation observed among regions was higher than the differentiation observed among populations within regions, however, most of the observed genetic variance was hosted within populations (> 99% according to the microsatellites in Datasets 1 and 2).

**Table 5:** Hierarchical AMOVA based on geographic regions for both sets of microsatellite data and Pan I

Loci	Source of variation	d.f	S.S.	Variance components (% of variation)	F-statistics
6 microsatellites	Among regions	6	118.39	0.014 (0.65%)	F_CT_ = 0.0065 (P < 0.0001)
(Dataset 1)	Among populations within regions	59	155.76	0.004 (0.19%)	F_SC_ = 0.0019 (P < 0.0001)
	Within populations	8626	18,279.78	2.119 (99.16%)	F_ST_ = 0.0084 (P < 0.0001)
15 microsatellites	Among regions	6	67.83	0.017 (0.36%)	F_CT_ = 0.0036 (*P* = 0.0001)
(Dataset 2)	Among populations within regions	11	60.39	0.006 (0.13%)	F_SC_ = 0.0013 (*P* = 0.0081)
	Within populations	2572	11,769.03	4.626 (99.51%)	F_ST_ = 0.0049 (P < 0.0001)
Pan I	Among regions	6	66.97	0.009 (8.29%)	F_CT_ = 0.0829 (P < 0.0001)
(Dataset 1)	Among populations within regions	59	33.34	0.004 (3.48%)	F_SC_ = 0.0379 (P < 0.0001)
	Within populations	8626	793.87	0.092 (88.24%)	F_ST_ = 0.1176 (P < 0.0001)

**Fig. 4:**
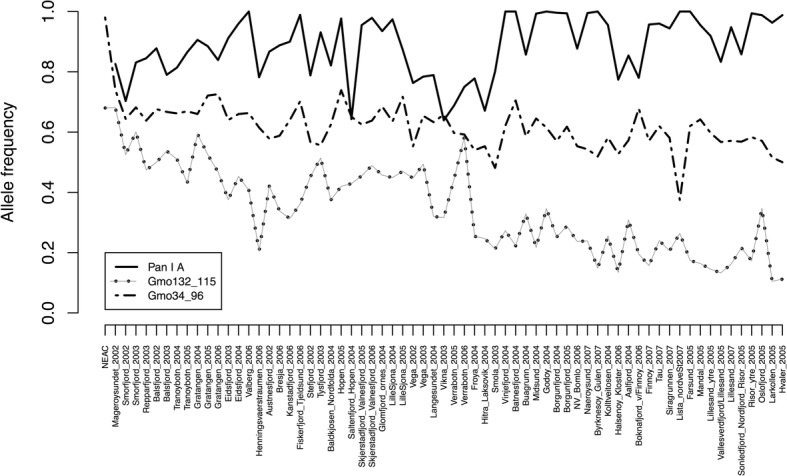
Frequency of the most common alleles within loci Gmo132, Gmo34 and Pan I in Dataset 1. Populations are ordered from north to south and the first one, called NEAC, consists of the 76 Pan I BB individuals with otolith types 4 and 5 that were purged from the dataset. Alleles Gmo132_115 and Gmo34_96 experienced a highly significant negative trend southwards (τ = − 0.626, *P* = 1.11 e-13, and τ = − 0.448, *P* = 1.17e-07, respectively), whereas for Pan I _A, this tendency was reversed (τ = 0.23, *P* = 0.007). The allele frequency for NEAC assessed in the Barents Sea was 0.745 for Gmo132_115, and 0.959 for Gmo34_96 [28]

Hierarchical AMOVAs per locus showed that the locus Gmo132 was the only one significant at all levels of division in both datasets (Table 6). This marker, which was depicted to be under directional selection by LOSITAN analysis, showed the highest differentiation within populations (average F_ST_ of 0.034). Locus Gmo34 showed significant F_CT_ and F_ST_ (average F_ST_ = 0.005) in all analyses. The extra set of nine microsatellites used in Dataset 2 showed one locus also significant at all levels (Gmo45), but also revealing a weak degree of structuring (average F_ST_ = 0.006). Interestingly, the most frequent alleles for both Gmo34 and Gmo132 displayed a strong and highly significant trend in the north-south gradient (Fig. 4). No such trend was observed for the most common alleles in Gmo45 (data not presented). Allele 96 from locus Gmo34 ranged from 0.739–0.375 (τ = − 0.448, *P* = 1.174 e-07) and allele 115 from locus Gmo132 ranged from 0.681–0.105 (τ = − 0.626, *P* = 1.111 e-13) in the direction north to south.

**Table 6:** Hierarchical AMOVA per locus based on geographic regions for both data sets

Data set	Locus	F_CT_	*P*-value	F_SC_	*P*-value	F_ST_	*P*-value
Dataset 1	Gmo2	0.0005	**0.0361**	0.0017	0.0659	0.0022	**0.0116**
	Gmo3	0.0008	0.0819	0.0020	0.0734	0.0028	**0.0171**
	Gmo34	0.0044	**0.0000**	0.0009	0.1809	0.0053	**0.0000**
	Gmo35	0.0001	0.4140	0.0008	0.2144	0.0009	0.1757
	***Gmo132***	0.0279	**0.0000**	0.0044	**0.0000**	0.0322	**0.0000**
	Tch11	0.0009	**0.0000**	0.0013	0.0517	0.0022	**0.0001**
Dataset 2	Gmo2	0.0001	0.4766	0.0024	0.0593	0.0026	**0.0293**
	Gmo3	0.0054	**0.0029**	0.0000	0.9186	0.0029	0.1193
	Gmo34	0.0057	**0.0028**	0.0000	0.8920	0.0036	**0.0344**
	Gmo35	0.0017	0.1859	0.0015	0.2201	0.0033	**0.0209**
	***Gmo132***	0.0249	**0.0003**	0.0054	**0.0003**	0.0302	**0.0000**
	Tch11	0.0005	0.2819	0.0011	0.2404	0.0016	0.0779
	GmoC18	0.0006	0.1656	0.0000	0.6458	0.0002	0.4323
	GmoC20	0.0008	0.1404	0.0000	0.7495	0.0001	0.5132
	GmoG13	0.0003	0.4134	0.0019	0.2641	0.0022	0.1730
	GmoG18	*******	*******	*******	*******	*******	*******
	GmoG25	*******	*******	*******	*******	*******	*******
	GmoG40	0.0017	0.1597	0.0020	0.3855	0.0037	0.1067
	GmoG43	0.0006	0.2317	0.0006	0.3822	0.0012	0.1233
	***GmoG45***	0.0034	**0.0027**	0.0027	**0.0012**	0.0061	**0.0000**
	Tch22	*******	*******	*******	*******	*******	*******

*P*-values in boldface type were significantly different from zero

**Fig. 5:**
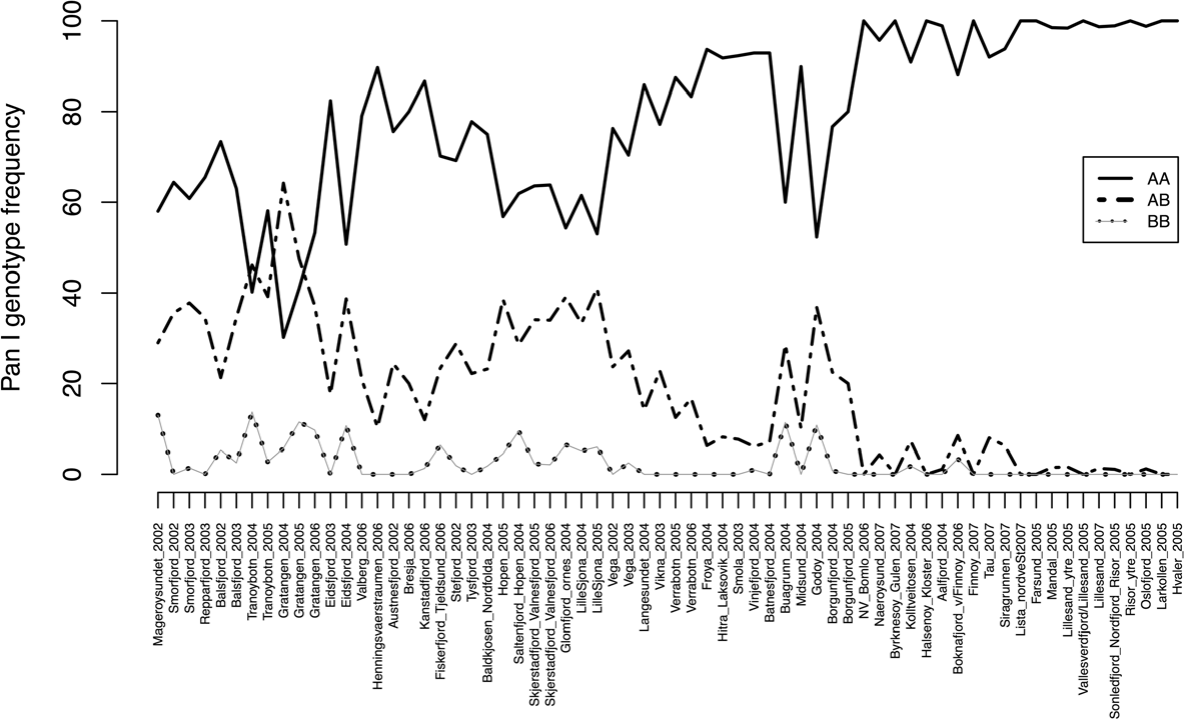
Frequency of the three different genotypes of locus Pan I in Dataset 1. Sites are ordered from north to south

Looking at the results for the Pan I locus, that was analysed for all samples, the three different genotypes displayed clearly distinct frequencies throughout the NCC samples (Fig. 5). Genotype AA was the most frequent and experienced an increasing trend towards the south, whereas genotype BB, present in low or very low frequencies in all populations, showed a decreasing southwards trend. The frequency of allele Pan I A revealed a negative trend towards the south (τ = 0.23, *P* = 0.0068, Fig. 4). Overall genetic structure for Pan I locus was highly significant (F_ST_ = 0.107, *P* < 0.0001) and hierarchical AMOVA was also significant at the three levels of grouping (Table 5). Likewise, pairwise F_ST_ for Pan I showed a highly significant north-south trend (τ = 0.871, *P* < 2.22 e-16).

**Fig. 6:**
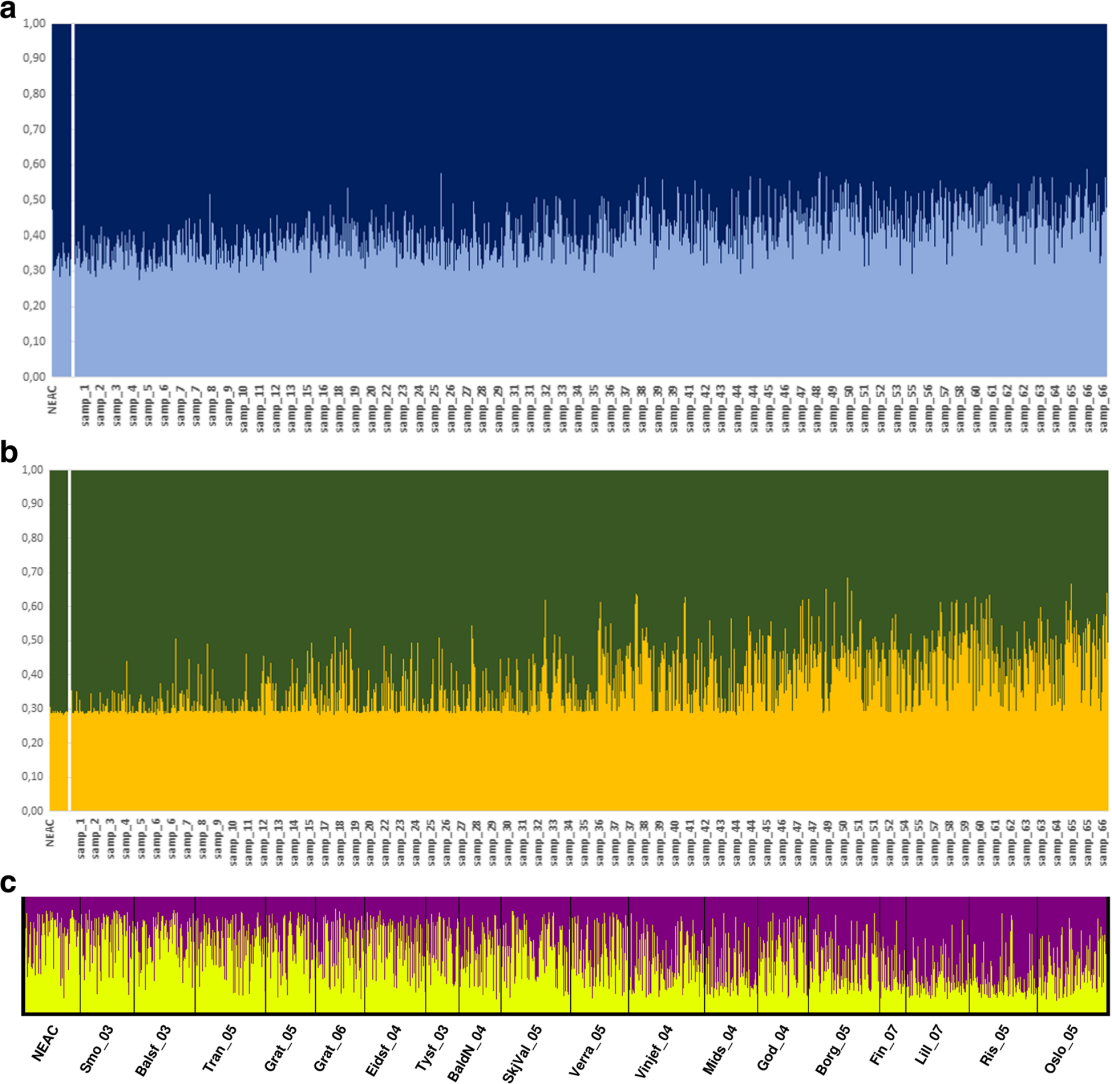
Bayesian clustering of cod samples. Inferred ancestry was assessed after clumping STRUCTURE runs where NEAC was used as an outgroup for NCC in the following datasets: Dataset 1 genotyped at (**a**) 6 microsatellites and (**b**) outlier loci Gmo32 and Gmo134; and (**c**) Dataset 2 genotyped at 15 loci. Sites were ordered from north to south as in Table 1

In order to investigate the potential admixture between both types of cod, NEAC was used as an outlier group for NCC in STRUCTURE. These analyses showed no clear clustering of populations in the full dataset genotyped at 6 microsatellites (Fig. 6a) in agreement with BARRIER (results not shown). However, the two outlier loci (Gmo32 and Gmo134) displayed a north-south gradient of admixture (Fig. 6b) whereby NCC showed greater genetic similarity to NEAC in the north than in the south. A similar trend could also be observed in the restricted dataset genotyped at 15 microsatellites (Fig. 6c).

## Discussion

Norway is the country with the largest remaining commercial catch of Atlantic cod, and this is the first study to investigate population genetic structure of coastal cod (NCC) along the entire Norwegian coastline. Following the analysis of > 4000 cod sampled from 55 locations, we obtained the following main results: 1. Statistically significant population genetic differentiation was revealed among most of the samples, 2. The observed genetic differentiation followed a pattern of isolation by distance along the north-south gradient, without any clear “breaks”, 3. No distinct change in genetic variation (i.e. allelic diversity or heterozygosity) was observed among the samples in the north-south gradient. Based upon these results, we conclude that NCC displays statistically significant population genetic structure along the Norwegian coastline. Consequently, these results demonstrate that the current management regime, dividing coastal cod in Norway into two management groups, one north and one south of 62°, represents an over-simplification of the true level of population genetic structure, and as such, requires re-evaluation.

As detailed in the introduction, Atlantic cod is one of the best-studied marine fishes, and a large number of population genetic studies have revealed a species displaying extensive population genetic structure (reviewed by [5–7]). While several of the newest studies in this species have utilised genomics methods to identify and characterise population genetic structure (e.g. [29, 32, 38, 41, 45]), our study based upon microsatellites and Pantophysin, has nevertheless made a significant contribution to our understanding of population genetic structure in this species, especially in Norway. First the biological, and then the management implications of our results are discussed below.

### Population genetic structure of NCC

The primary aim of the present study was to investigate population genetic differentiation among coastal cod sampled from the entire Norwegian coastline, which stretches over 2500 km from the south in Oslofjord, to the north in Porsangerfjord (Fig. 1). In some areas of Norway, in particular the north, NEAC are found using the same spawning areas as NCC [80, 81]. Consequently, some NEAC were inadvertently collected during the sampling process, especially in the north (Table 1). In order to eliminate the potential bias caused by NEAC in the present analysis of NCC population genetic structure, we used otolith categories 4 and 5 to exclude any potential NEAC from our biological samples prior to genetic analysis [58].

The Pan I BB genotype is nearly fixed for NEAC [26], and is almost completely diagnostic between NCC and NEAC [18, 26, 27]. In addition, Pan I genotype and otolith type both clearly differentiate between NEAC and NCC [27, 82]. Despite purging otolith category 4 and 5 individuals from the current data set, 104 fish displaying an otolith structure typical for coastal cod (i.e., category 1–2) with the Pan I BB homozygote genotype (i.e. characteristic for NEAC) remained. Importantly however, and in the context of the present study, both including and excluding these 104 BB homozygote individuals did not influence the overall picture of population genetic structure. Therefore, it is concluded that we have effectively excluded NEAC from these analyses, and that the differences reported here primarily reflect genetic differences between NCC populations along the Norwegian coastline. Given that genetic differentiation did not show any clear genetic groupings, nor clear breaks in population connectivity, we suggest that NCC populations belong in a genetic gradient characterised by isolation by distance. This pattern is potentially driven by the generally limited migratory behaviour of NCC as has been seen in tagging experiments ([24, 81, 83], but see [84]), spawning site fidelity [41, 85], and potentially retention of eggs in certain fjord areas [83, 86].

A distinct north-south cline in the frequency of the allele 115 for the microsatellite Gmo132 was observed here (Fig. 4). This pattern may be explained using two different scenarios: 1) Since this genetic marker has been tagged as an outlier in previous genetic analyses [26, 87], the observed north-south cline at this locus might have been shaped under the influence of environmental factors. In our data however, significant allele frequency differences for this locus also existed among samples within the same region, thus it is unlikely that selection alone has created the observed north-south allelic cline (see [28]). 2) Although NEAC were removed from most of the analyses of the present study, this does not preclude the possibility that genetic introgression and admixture between NEAC and NCC contributes to the observed pattern of population genetic structure in NCC. Supporting this suggestion is the fact that the frequency of the 115 allele in Gmo132, in all of the northern samples investigated here, displayed a frequency around 0.5–0.6, which is very similar to the frequency of this allele in NEAC [~ 0.7 28]. Therefore, admixture between NEAC and NCC, primarily in the north and following a decreasing gradient of presence towards the south, may contribute to the pattern of population genetic structure observed in NCC. This suggestion is consistent with simulations [88] illustrating that introgression from a genetically distinct source (e.g., NEAC) may generate gradients in allele frequencies along a geographic axis originating at the edge of the contact zone (i.e., north in the current study). These “tails of introgression” may extend to large distances beyond the contact zone itself (i.e., towards the south in the cod case). Importantly, the gradient only appears where dispersal and gene-flow are spatially limited. In our case, the limited gene-flow among NCC populations in Norway results in a gradient in the allele frequency, which starts in the north due to introgression of NEAC.

Population genetic differentiation was observed among samples of NCC throughout all regions of Norway, including among samples from southern Norway where NEAC was hardly present (as determined either by Pan I genotype or otolith category). In the far southern region, it is unlikely that NEAC has played a direct role in shaping the evolutionary relationships among NCC populations (although it is possible that the tail of potential introgression from NEAC in the north could still extend to the far south and thus play a minor role). However, the interaction between migratory North Sea cod and NCC populations in the south of Norway also remains a potential source of influence on NCC structure in this region [89]. Clearly, the evolutionary relationship between NEAC and NCC, and North Sea cod and NCC, needs to be resolved in order to evaluate the degree of influence migratory ecotypes have on the population genetic structure of NCC throughout Norway.

Current evidence suggests that the long-distance migratory “ecotype” of Atlantic cod, including NEAC, was derived from the stationary “ecotype” [29], and that this divergence occurred prior to the split between the Northeast and Northwest cod populations [90], which has been estimated to have occurred approximately 100,000 years ago [91, 92]. As stated above, NEAC and NCC exhibit large genetic and genomic differences between them, and while several potential mechanisms have been proposed [80, 93, 94], the ecological processes leading to and maintaining segregation of these two ecotypes, and the degree of genetic exchange between them in both time and space, remain unresolved. The (~ 2%) BB homozygotes observed in the present data set, that displayed an otolith structure typical for coastal cod, could fit in one of these scenarios: 1 – “true” NEAC that for some reason did not migrate to and from the Barents sea as is characteristic for NEAC, 2 – hybrid and or admixed individuals between NEAC and NCC that did not migrate to and from the Barents sea, possibly due to only carrying one set of chromosomes with the “inverted supergene” and therefore displaying reduced propensity for long-distance migration, 3 – “true” NCC cod that display the BB genotype as the B allele is observed in NCC although at low frequencies. Quantifying admixture between NEAC and NCC remains a challenge that even recent papers focussing on the genome-wide differences between NEAC and NCC have not completely resolved [29, 32]. Pan I lies within one of the inverted regions of chromosome 1, which together with inversions in linkage groups 2 and 7 are responsible for nearly all of the genomic divergence between NEAC and NCC [29, 32]. As these inversions block recombination in their respective locations on the genome for NEAC, this means that if NEAC and NCC hybridise, their offspring will contain one copy of the inverted parts of linkage groups 1, 2 and 7, and one copy of the ancestral collinear form. We suggest that a genomic analysis of BB homozygotes displaying otolith categories 1–2 may provide an important resource in resolving the issue of hybridisation between NEAC and NCC, and the degree to which this may or may not influence population genetic structure of NCC [95]. Finally, the discrepancy in the allele frequencies in Pan I (linkage group 1) and Gmo132 (linkage group 7) reported between NEAC and NCC along the north-south gradient require further investigation.

### Management implications

Applying the same management strategy to multiple populations or stock components that vary in their abundance and/or resilience to exploitation inevitably results in overfishing and likely collapse of the weaker component [96, 97]. The extent of population genetic structure revealed in the present study, irrespective if it is influenced by a gradient of NEAC admixture or not, and divergent selective forces or not, strongly suggests that the current division of coastal cod in Norway, above and below 62° north, will not be sufficient to ensure sustainable management of NCC throughout Norway. Our data clearly illustrate population genetic structure within all areas of Norway, and as such, needs to be taken into consideration when renewing management plans. This conclusion is supported by earlier studies of population genetic structure in the south of Norway, where temporally-stable population genetic differentiation has been observed between neighbouring fjords over relatively small distances, effectively demonstrating limited connectivity at least between some fjord systems [24, 98, 99]. Additionally, earlier data from the north of Norway, revealing genetic differences between coastal cod sampled in fjords, supports the main results from the present study [14, 19, 26, 28]. It is nevertheless acknowledged that the lack of any clear “breaks” in population connectivity along the Norwegian coastline means that identifying appropriate management units is challenging.

An additional management contribution from the present analyses is the apparent lack of any clear differences in genetic variation, as reported by numbers of alleles, allelic richness, heterozygosity or the effective population size (Fig. 2-4; Additional file 3), in the north-south gradient. In all areas of Norway, NCC has been documented in decline since 1990s, to which overfishing has probably played a significant role. However, in the southern regions, populations also appear to be influenced by climate-driven recruitment challenges as has been illustrated by the beach-seine survey conducted in this region since 1919 [100]. Despite this, based upon the samples analysed here, there are no indications of severe genetic bottlenecks in any of the samples, and no clear differences in genetic diversity estimates between the north and south.

## Conclusions

Norwegian coastal cod displays statistically significant population genetic structure along the Norwegian coastline, which follows a genetic gradient characterised by isolation by distance. Although not fully resolved, we suggest that genetic introgression and admixture between NEAC and NCC, most in the north and least in the south, together with limited gene-flow among NCC populations, may contribute to the observed population genetic structure of NCC. In turn, we conclude that the current management regime in place, dividing coastal cod in Norway into two management groups, north and south of 62°, represents an over-simplification of the true level of population genetic structure, and as such, requires re-evaluation.

## Additional files

Additional file 1: **Figure S1.** Ho and uHe per sampling site for **(a)** Dataset 1 (66 samples genotyped at 6 microsatellite markers) and **(b)** Dataset 2 (18 samples genotyped at 15 microsatellite markers). Locations are ordered from north to south along the coastline. In graph a), Ho and uHe experienced significantly increasing N-S trends (τ = 0.363, *P* = 1.705 e-05, and τ = 0.570, *P* < 2.22 e-16, respectively). Unlikewise, in graph b), neither Ho nor uHe showed any kind of N-S trend (τ = 0.281, *P* = 0.11164 and τ = 0.21, *P* = 0.23997, respectively). (ZIP 1059 kb)

Additional file 2: **Table S1.** Genetic differentiation at Dataset 1 (66 samples genotyped at 6 microsatellite markers): Pairwise F_ST_ are shown in the lower diagonal whereas *P*-values computed after 10,000 permutations are shown in the lower diagonal. (XLSX 77 kb)

Additional file 3: **Raw data.** Genotypes for Dataset 1 (66 samples genotyped at 6 microsatellites) and Dataset 2 (18 samples genotyped at 15 microsatellites). Pan I genotypes, and otolith information is provided for each individual as well. (XLSX 563 kb)

## Abbreviations

AR: Allele Richness
F_CT_: F-statistics among groups/regions
F_SC_: F-statistics within populations
F_ST_: a commonly used measure of population differentiation due to genetic structure
H_O_: observed heterozygosity
LD: Linkage Disequilibrium
NCC: Norwegian Coastal Cod
NEAC: North East Arctic Cod
NSC: North Sea Cod
Pan I: Panthophysin I
PCR: Polymerase Chain Reaction
SNP(s): Single Nucleotide Polymorphism(s)
uHe: unbiased expected heterozygosity
