# Supplementary figures and images for: Analysis of coastal cod (Gadus morhua L.) sampled on spawning sites reveals a genetic gradient throughout Norway’s coastline
Source: BMC Genet. 2018 Jul 9;19:42. doi: 10.1186/s12863-018-0625-8 (PMC6036686; doi:10.1186/s12863-018-0625-8)

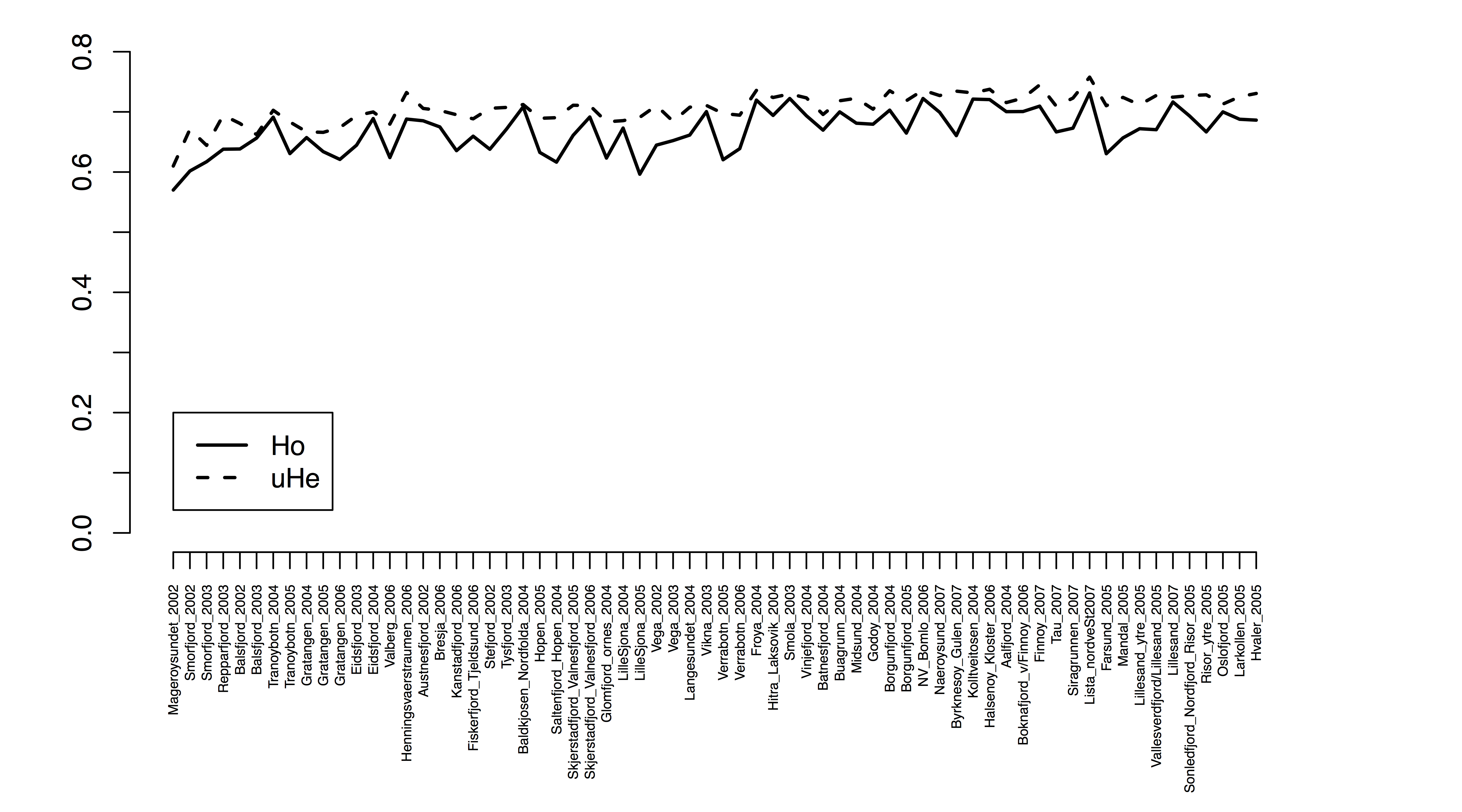

Supplement: Supplementary file 1 — Figure S1. Ho and uHe per sampling site for (a) Dataset 1 (66 samples genotyped at 6 microsatellite markers) and (b) Dataset 2 (18 samples genotyped at 15 microsatellite markers). Locations are ordered from north to south along the coastline. In graph a), Ho and uHe experienced significantly increasing N-S trends (τ = 0.363, P = 1.705 e-05, and τ = 0.570, P < 2.22 e-16, respectively). Unlikewise, in graph b), neither Ho nor uHe showed any kind of N-S trend (τ = 0.281, P = 0.11164 and τ = 0.21, P = 0.23997, respectively). (ZIP 1059 kb) [file 12863_2018_625_MOESM1_ESM.zip › Fig S1a.tif]

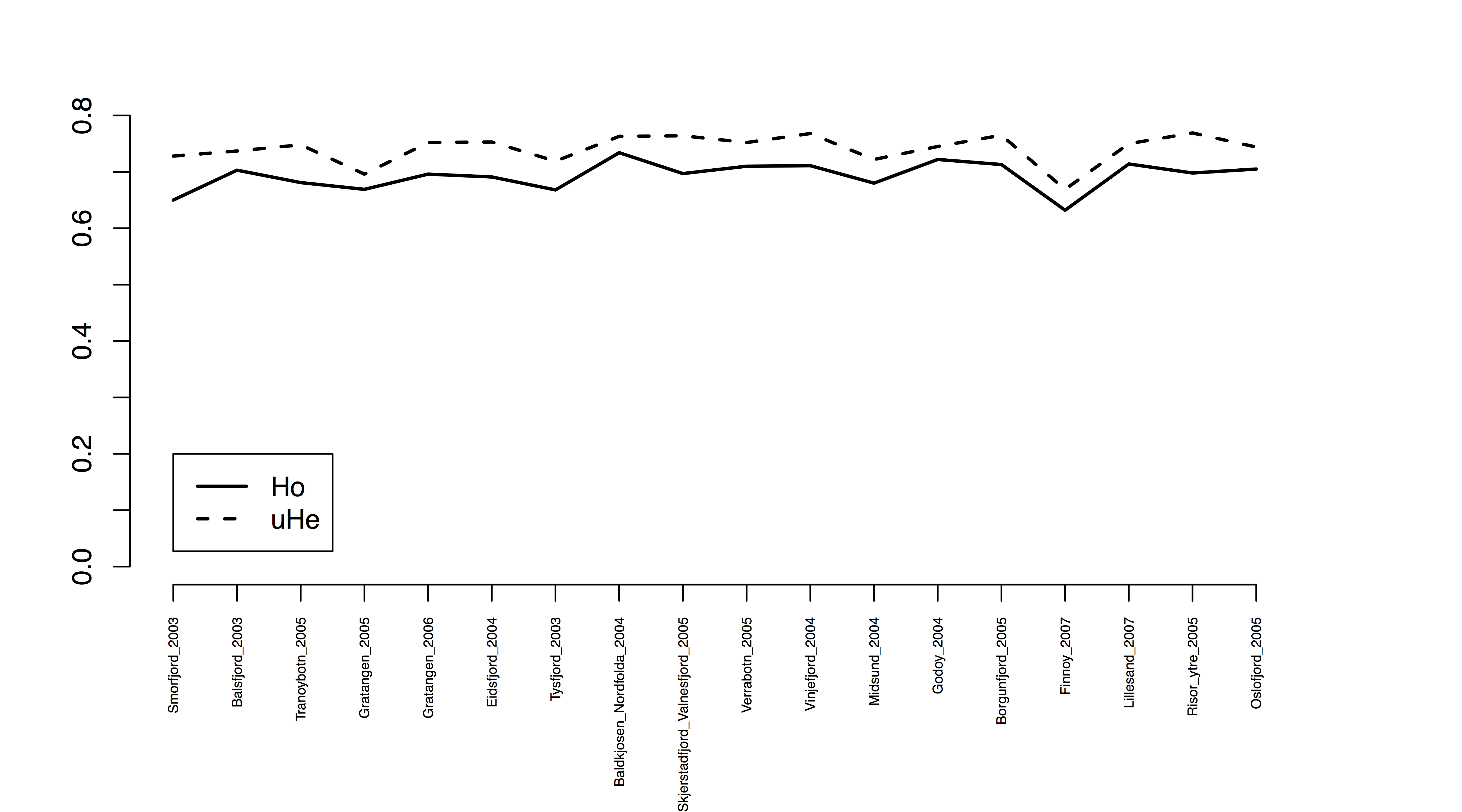

Supplement: Supplementary file 1 — Figure S1. Ho and uHe per sampling site for (a) Dataset 1 (66 samples genotyped at 6 microsatellite markers) and (b) Dataset 2 (18 samples genotyped at 15 microsatellite markers). Locations are ordered from north to south along the coastline. In graph a), Ho and uHe experienced significantly increasing N-S trends (τ = 0.363, P = 1.705 e-05, and τ = 0.570, P < 2.22 e-16, respectively). Unlikewise, in graph b), neither Ho nor uHe showed any kind of N-S trend (τ = 0.281, P = 0.11164 and τ = 0.21, P = 0.23997, respectively). (ZIP 1059 kb) [file 12863_2018_625_MOESM1_ESM.zip › Fig S1b.tif]
